# Baltimore Medical Association

**Published:** 1884-03-20

**Authors:** 


### BALTIMORE MEDICAL ASSOCIATION.

### STATED MEETING HELD DECEMBER 10TH, 1883.

The Association was called to order at the usual hour and, in the absence of the President and Vice-Presidents, Dr. C. H. Jones was called to the chair.

HEMORRHAGE AFTER TONSILLOTOMY.—Dr. Perkins reported a case of hemorrhage after removal of the tonsils in a girl æt. 17. It was the second case of the sort he had had. The hemorrhage occurred on the third day after operation, was checked without difficulty, and there has been no recurrence of it.

OBSTINATE VOMITING FROM PREGNANCY.—Dr. Gibbons reported a case of obstinate vomiting. He was called to see the patient (a young woman two months advanced in pregnancy) about six weeks ago. All the usual remedies were tried in vain, including the last one recommended, pop-corn. Relief has finally been obtained by a combination of vichy water and sherry wine. Dr. A. Atkinson had succeeded with a mixture of creasote, gtt. j, and infusion of columbo, ℥ ss, after failure of other remedies. The Chairman suggested bromide of potassium and hydrocyanic acid. Dr. J. T. Smith had very little confidence in any specific remedy; the vomiting ceases of itself and the last thing given gets the credit. Dr. Kemp, Jr., referred to a very severe case, lasting from the fifth week to the end of the eighth month. Dry cold applied to the abdomen gave more relief to the patient than anything else. The patient was compelled to remain recumbent all the time, in order to obtain relief. Dr. Gibbons said dry cold was tried without effect on his patient. She vomited both lying down and when sitting. Dr. Taneyhill had applied Lugol's solution to the os uteri in three cases, with relief. Dr. Reinhart had found tinct. of nux vomica and citric acid solution effectual. Dr. Scarff said there was no specific for it. Hydrate of chloral relieved a lady in one pregnancy, but failed in the next.

AVENA SATIVA AS A SEDATIVE.—Dr. A. Atkinson spoke of the excellent effect he had obtained in a patient who had been drinking from the tinct. of avena sativa. It had acted better than chloral, bromide of potash or opium. Dr. King had obtained marked sedative effects from the same drug in 40 drop doses.

CHLORAL IN PUERPERAL ECLAMPSIA.—Dr. Taneyhill referred to the good effects obtained in a patient, suffering from puerperal convulsions, from rectal injections of hydrate of chloral in 30 grain doses every two hours. The convulsions began about 30 to 35 minutes after confinement. The urine coagulated completely in the test tube, on the application of tests for albumen.

PRURITUS ANI.—Dr. A. Atkinson opened the regular discussion on this subject. He attributed the affection to an eczema, which produces an annoying itching, especially marked at night, when the sufferer is in bed. It may be produced by ascarides and is promoted by constipation, diarrhœa, alcohol, coffee, tea, sweets, feather beds, smoking, late suppers, etc. The use of water locally seems to intensify it. The treatment is partly deducible from the above and includes saline cathartics, sulphur and cream of tartar, the saline and sulphur mineral waters, exercise to fatigue, hot water injections, a large dose of bromide at bed-time, the aqueous extract of opium in suppositories, application to the anus of a strong solution of boracic acid (which was strongly recommended), the use of yellow wax or mercurial ointment, abstention from rubbing and the attention to diet.

The chairman had used iodoform with relief in one case.

Dr. Gibbons said the application of fluid extract of gelsemium will relieve itching.

Dr. Scharff employs a solution of iodoform in chloroform.

Dr. Perkins thought that the virtues of iodoform are often impaired by the attempts to overcome its odor, and that the disagreeability of the latter is exaggerated.

Dr. Taneyhill recommended the use of Parr's soap immediately after stool and at night, followed by the application of oleate of mercury (10 pr. ct.). He would guarantee a cure by this treatment in three weeks. Of eleven cases which he had thus treated, all were cured.—*Southern Clinic.*

---

**Migraine.**—Dr. Morton (Med. Gaz.) says: "The most satisfactory single remedy for the form of migraine in which there is angio-spasm is the bromide of sodium. Drachm doses are to be given at every onset, and repeated in an hour and a half, if the attack does not cease. This will seldom fail in cutting short an attack. In addition, the same remedy should be given in ten-grain doses three times a day, for a period of from three to six months. The sodium salt does not depress the whole economy to anything like the extent the potassium salt does. Cod-liver oil and iron may be given in conjunction with the sodium bromide. The theoretical objection to the use of an agent believed to lessen the amount of blood in the brain, in a condition in which there is supposed to be anæmia of the affected side, is answered by the paradoxical assertion that in the spastic form of migraine the bromide probably increases the quantity of blood in the anæmic area by depressing the function of the vaso-motor system. Nitro-glycerina and nitrite of amyl, presumably from their power to control spasm, are very useful in the spastic form of migraine. The effects of nitrite of amyl are probably the more transient of the two. Nitro-glycerine is often of remarkable efficacy. A one-per-cent. solution may be given in doses gradually increased from one drop to five drops, three times daily. This agent is believed to be decomposed, in the presence of an alkali, with the production of nitrous acid. This decomposition may take place in the stomach, if it be alkaline; otherwise, and more slowly, in the blood. Nitro-glycerine is best given after the meals. The bromide of sodium may be given with advantage, at the same time, before meals.

